# Defining and Developing a Global Public Health Course for Public Health Graduates

**DOI:** 10.3389/fpubh.2015.00166

**Published:** 2015-07-01

**Authors:** Rajendra Karkee, Jude Comfort, Helman Alfonso

**Affiliations:** ^1^BP Koirala Institute of Health Sciences, School of Public Health and Community Medicine, Dharan, Nepal; ^2^School of Public Health, Curtin University, Perth, WA, Australia

**Keywords:** global health, competencies, course, teaching

## Abstract

Global public health is increasingly being seen as a speciality field within the university education of public health. However, the exact meaning of global public health is still unclear, resulting in varied curricula and teaching units among universities. The contextual differences between high- and low- and middle-income countries, and the process of globalization need to be taken into account while developing any global public health course. Global public health and public health are not separable and global public health often appears as an extension of public health in the era of globalization and interdependence. Though global public health is readily understood as health of global population, it is mainly practiced as health problems and their solutions set within low- and middle-income countries. Additional specialist competencies relevant to the context of low- and middle-income countries are needed to work in this field. Although there can be a long list of competencies relevant to this broad topic, available literature suggests that knowledge and skills related with ethics and vulnerable groups/issues; globalization and its impact on health; disease burden; culture, society, and politics; and management are important.

## Introduction

The last century has seen the world becoming more and more a “global village” with rapid globalization and complex interdependence (1). Production and consumption patterns of goods and services have made nations more interdependent to each other. Mobility of people, flow of resources and technologies, and transmission of diseases and information across borders have become instant. The flow can occur in either direction from high to low- and middle-income countries and vice versa. Consequently, public health issues of any country can become global health risks and take global attention. However, a lot of activities of bilateral, multilateral, and non-governmental organizations have been directed to improve the health in low- and middle-income countries in the name of “global health.”

In fact, global health has been fashionable, with the establishment of centers or institutes and restructuring of academic programs in universities to encompass this area (1–6). The global health domain is often included by donor governments in their foreign aid policy or by large funders as their “aid” (7, 8). Global public health is now a growing specialist field within the university education of public health. Graduate degree programs in global public health are flourishing in universities of high-income countries (9). There is also an argument that health institutions and universities of high-income countries have a greater role and responsibility in global health training and global health improvement. However, the exact meaning of global public health remains unclear, thereby producing varied curricula and teaching units in university courses. Further, the terms “global health” and “global public health” are often used interchangeably. These two terms can be differentiated. Owing to the important role of health in Millennium Development Goals and post-2015 Sustainable Development Goals including climate change and food security, a coherent idea of global health is being expected (10). The purpose of this article is to discuss what global public health means and what health competencies should be included at a graduate study level.

## Discussion

### Global public health and public health

There have been attempts to differentiate between “public health,” “international health,” and “global health.” Global health is derived from public health and international health, which, in turn, evolved from hygiene and tropical medicine (11). Public health is related to the populations of a particular country or community with a focus on prevention. In fact, public health can be defined as the organized efforts of community to prevent disease, promote health, and prolong life among the population as a whole. International health is concerned with health issues of low- and middle-income countries embracing prevention and clinical care and relying on bilateral cooperation and technology (11, 12). Global health focuses on issues that transcend national boundaries affecting population worldwide as a result of globalization and approach requires global cooperation (10, 13). Global health is seen as highly interdisciplinary encompassing prevention, treatment, and care with involvement of several disciplines including social and behavioral sciences, law, economics, history, engineering, biomedical and environmental science, and public policy (14).

However, such distinction of global health, international health, and public health has been contested by others. Principally, public health and global health cannot be separable because both are based on same foundation and tenets such that “global health is public health” (14). This means that global health can be thought of either as parallel to the public health or as a speciality within public health. In fact, graduate academic programs relating to global public health have been termed in different ways in universities around the world: master of public health in global health, master of global health, master in international public health, and master in international health. However, the term “global health” is becoming more popular (15). Since global health can encompass all health-related activities of different disciplines, for example, nursing, medicine, or sociology, it may be more suitable and consistent to use the term “global public health” to limit global health within public health discipline.

### Delineating global public health

In the media, in lay and scientific literature, and in major initiatives, all activities in public and allied health targeted in international aspects, mainly in low- and middle-income countries, are being defined as global health, resulting in a clear lack of agreed global health competencies and varied curricula among universities (16). Within such broad and complex ideas of global health, a public health school or program is left to define its own activities and priorities that it considers as global public health (preferably, against its set mission/objectives, resources, and approaches). In defining the basic outline of study in global public health, two factors are important: first, the apparent difference of the “context” between high-income countries and low- and middle-income countries in terms of social structure, health system, disease burden, etc. (creating the so-called North-South divide) and second, the interdependence and globalization of health.

Though high- and low-income countries are becoming more alike in some disease patterns including non-communicable, mental disorders, and injuries, there are huge disparities in many health and social indicators including maternal and infant mortality, life expectancy, health care coverage, sanitation, and human resources. The low-income countries are typically characterized with inadequate human and material resources, poor governance and management, larger portion of population under the age of 15; weak health system, and high maternal and child mortality. These differences in context and health outcomes obviously necessitate a different orientation of public health training and approaches. Another important concept to be included in all public health courses is globalization and its impact on health. Globalization has now become one of determinants of health (10). Not only transmissions of diseases across borders are a threat but also economic policies, politics, trade treaties, expansion of multinational companies, and consumption of foods affect health worldwide. Addressing of these global health problems is not within the control of a single nation but need global health governance (13). Thus, global public health requires a critical understanding of the drivers that have resulted in such global health risks, especially different health outcomes between high- and low-income countries.

Global public health is considered as health of global population, it is, however, not surprising that many academic institutions in high-income countries have ultimately focused the training with emphasis on health problems and their solutions in low- and middle-income countries. This has left the global public health training in universities of high-income countries mainly as public health training for developing countries. This raises the question of what constitutes global public health as a speciality in universities of low- and middle-income countries. One possibility is that universities in low- and middle-income countries may be interested in globalization aspects of health and health care organization in high-income countries. However, in our search of public health courses in South Asian Countries (Afghanistan, Bangladesh, Bhutan, India, Maldives, Nepal, Pakistan, and Sri Lanka), we did not find any degree or speciality that has been termed as global health or global public health.

### Specifying global public health knowledge and skills

Since global public health is necessarily an extension of public health in a global context, additional competencies mainly relevant for low- and middle-income countries are required for someone who wishes to specialize in global public health (17, 18). Thus, it is first necessary for a School of Public Health to specify basic public health competencies and then additional competencies relevant in global public health specialization. Core public health competencies have been developed in North America (18, 19), Europe (20), Asia-Pacific, and Australia (21) by related agencies but not all specify on global health competencies. Similarly, competencies for graduate level public health training in India, China, and other low- and middle-income countries have also been defined (17, 22, 23). Basic public health knowledge and skills common to all of the above include quantitative methods (epidemiology and biostatistics); sociological, environmental, and behavioral base for public health; and management and policy skills (Table S1 in Supplementary Material). Building on these basic competencies, Schools of Public Health can develop global public health competencies needed to specialize in global public health training.

There is little literature published on the knowledge and skills for global public health. The Association of School of Public Health in USA has developed global health competencies, which build upon its Master in Public Health core competencies model (19). Ablah and colleagues describe these global health competencies in detail (9). They acknowledge the role of diversity of infrastructural, socio-cultural, and political environments that determine the global health competencies scenario and describe 36 competencies under 7 domains for global health competency master program in USA. The seven domains are (1) capacity strengthening, (2) collaborating and partnering, (3) ethical reasoning and professional practice, (4) health equity and social justice, (5) program management, (6) socio-cultural and political awareness, and (7) strategic analysis.

Pfeiffer and colleagues conducted 26 in-depth interviews with global health leaders to define the education and training for twenty-first century competency-based Global Health master’s curriculum for the University of Washington’s Department of Global Health. After analysis of interviewees’ opinion, the authors suggested five competencies for Global Health curriculum: (1) knowledge of social, economic, and environmental determinants of health; (2) knowledge of the architecture and levers of health and health-relevant systems and health service delivery; (3) skills in epidemiology and in monitoring and evaluation; (4) capacity to manage and lead; and (5) skills in policy analysis and development (24). Cole and colleagues summarizes competencies in global health and research, which were defined at the Canadian Public Health Association’s Centennial Scientific Conference in June 2010 (18). They have proposed complementary competencies for global health practice and global health research and highlight knowledge and skills for global health as “north–south power dynamics, linkages between local and global health problems, and the roles of international organizations.” They suggest that graduates must be able to work responsibly in low-resource settings, foster self-determination in a world, which has power differentials, engage in dialogue with stakeholders globally, and possesses cross-cultural communication skills.

Akbar and colleagues identified competencies for Australian health professionals working in International Health through a short survey. They emphasized that high level of cultural, interpersonal, and team-work competencies are of great value while working in international settings (25).

**Figure 1:**
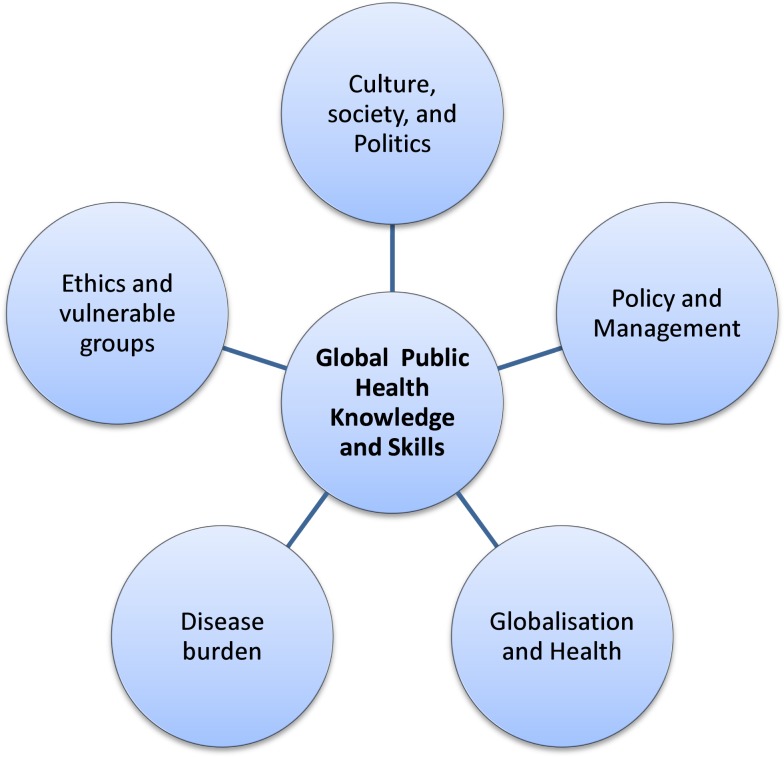
**Areas relevant to global public health knowledge and skills**.

Negin and colleagues argued that teaching “health systems in low- and middle-income countries” should be included in global health and argued that global health institutions have a responsibility to support health systems in low- and middle-income countries (26). Most of the large global health players (WHO, World Bank, etc.) put health systems at the centre of their health strategies and global health policy. Health systems are concerned with cost, quality, accessibility, delivery, organization, and financing of health care to improve health outcomes. Though there can be a long list of knowledge and skills relevant to broad global public health, the available literature indicates that there are some common important competencies which a graduate specializing in global public health requires to work in international settings. Figure 1 lists five emerging areas relevant to global public health knowledge and skills and Table 1 lists units associated with those areas to consider for inclusion in any global public health speciality course.

**Table 1:** **Relevant units and rationale for inclusion in a global public health course**.

Units	Rationale	Potential content
Primary health care and health promotion in low- and middle-income countries	Primary health care remains a core strategy to provide health care and a tool for health and social development. It also incorporates preventive health approaches through health promotion strategies	History and importance of primary health care Principles and strategies of primary health care Organization of primary health care and district health system Health promotion strategies in low- and middle-income countries

Comparative health system	The organization, cost, and accessibility of health systems impact on resulting health care. Cross-country comparison gives important insight for so-called universal coverage, a policy advocated by WHO. Important for those who work in government health systems	Structure and purpose of health system Health system functions Major health system organization models with emphasis in models of health system financing; cross-country comparison Dilemma of health care reform and public private mix

Program/project development, management, and evaluation	A lot of international donations, private sector, and national health services are conducted through project delivery. Non-governmental organizations are important employers for global public health graduates	Overview of project cycle management process Problem identification and stakeholder analysis Project planning with the logical framework approach Project implementation: costing, scheduling, and risk management Project evaluation and monitoring

Management, leadership, teamwork	Collaborating, partnerships, leaderships, and teamwork are necessary to work in national and international settings. Almost every organization requires their employees to have these skills	Management and leadership skills Interpersonal relationships and team work Management and organizational structural models

Globalization and health	International multilateral and bilateral organizations impact on global health including national health policy; movement of health workforce and disease agents; international response and regulations, etc., are “must” know for every public health graduate	Definition of globalization Health impacts of globalization both positive and negative Key actors in global health: multilateral and bilateral Future globalization challenges such as climate change Relationship to sustainability goals

Maternal and child health	Maternal and child health status is still unacceptably poor in most low- and middle-income countries. It is an area of employment as well as essential contribution to improvement of global health. Maternal and child health are both listed in the Millennium Development Goals and will also impact on Sustainable Development Goals	Foundation of maternal and child health, maternal, and child morbidity and mortality Maternal and child health care services and their utilization Strategies and components of safe motherhood program and child health program

Global disease burden	Epidemiology of communicable and non-communicable disease with special importance on HIV, malaria, and tuberculosis and the epidemiological transition; this will give a landscape of disease problems in rich and poor countries	Current global health issues and disease burden examining both communicable and non-communicable diseases Epidemiological transition and its challenges, with reference to aging population Future global disease burden and its challenges

Culture, social system, social development, and health	This is particularly important for graduates from developed countries or in situations of cross-cultural work	Need to understand importance of cultural setting when addressing health issues Community participation and stakeholders analysis Ecological framework for understanding micro and macro settings and interconnectedness

## Conclusion

Since global public health is an extension of public health, after having basic public health competencies, additional competencies especially relevant to the context of low- and middle-income countries are needed to work in this field. Available literature suggests knowledge and skills related with ethics and vulnerable groups/issues; globalization and its impact on health; disease burden; culture, society, and politics; and management are important to include in any global public health speciality if we are to produce graduates able to tackle the issues of global public health equity as reinforced in such documents as the Millennium Development goals and the Sustainable Development Goals.

## Author Contributions

RK conceived the idea of the debate, reviewed literature, and drafted the article. JC and HA helped in revision and interpretation of the literature. All the three authors read and approved the final version of the article.

## Supplementary Material

The Supplementary Material for this article can be found online at http://journal.frontiersin.org/article/10.3389/fpubh.2015.00166

## Conflict of Interest Statement

The authors declare that the research was conducted in the absence of any commercial or financial relationships that could be constructed as a potential conflict of interest.
