# Supplementary material for: Defining and Developing a Global Public Health Course for Public Health Graduates
Source: Front Public Health. 2015 Jul 1;3:166. doi: 10.3389/fpubh.2015.00166 (PMC4486750; doi:10.3389/fpubh.2015.00166)
Supplement: Supplementary file 1 [file table_1.docx]

**Supplementary Table 1** Core public health competencies

| **Auspicing association** | **Core public health competencies** |
| --- | --- |
| Associations of School of Public Health, USA | **Core**: Biostatistics, Environmental Health Sciences, Epidemiology, Health Policy and Management, Social and Behavioural Health  **Additional:**  Interdisciplinary Core competencies: Communication and Informatics, Diversity and Culture, Leadership, Public Health Biology, Professionalism, Program Planning, and Systems Thinking |
| Association of School of Public Health, Europe | Methods in public health ( Epidemiology, Demography, Biostatistics, Quantitative research methods, Qualitative research methods, Sociology, Anthropology); Population Health and its social and economic determinants; Population health and its material (environmental determinants); Health policy, economics, organisational theory, and Management; Health promotion (health education, health protection and disease prevention); Ethics |
| Canadian Public Health Agency | Public Health sciences; Assessment and Analysis; Policy and Program Planning; Implementation and evaluation; Partnerships, collaboration and advocacy; Diversity and inclusiveness; Communication; Leadership |
| Australian Network of Academic Public Health Institutions | Health monitoring and Surveillance; Disease prevention and Control; Health protection; Health Promotion; Health policy planning and management |
| Asia Pacific Consortium for Public Health | Biostatistics, Epidemiology, Health policy and management, Social and Behavioural Science, and Environmental sciences |
